# Analysis and Properties of the Gastric Fluid

**Published:** 1845-05

**Authors:** 


*Analysis and properties of the gastric fluid.*—The most remarkable results of M. Blondlot's investigations relate to the composition of the gastric fluid, and different as his conclusions may be from those usually received, yet the large quantity of fluid he was enabled to collect in a purer state than any one hitherto has collected it, entitles his account to every consideration. He very carefully distilled on a sand-bath 3875 grains of pure gastric fluid obtained after feeding his dog with raw meat; he repeated the distillation, and repeated the whole experiment, several times, with the gastric fluid of other animals as well as of the same dog, and the constant result was, that the product of the distillation did not once exhibit the slightest acid reaction; but the residue in the retort was always strongly acid. It was thus proved that the acid of the gastric fluid cannot be either the hydrochloric or the acetic, for both these are volatile at the boiling point of water, and would have distilled over.

The general conclusion of his analysis is, that the gastric fluid is composed of ninety-nine parts of water, with one part of super-phosphate of lime, super-phosphate of ammonia, chloride of sodium, mucus, an aromatic, and a peculiar, principle.

Similar results were obtained from the analysis of the gastric fluid of several animals.

Besides these experiments concerning the chemical properties of the gastric fluid, M. Blondlot relates others, which add to the evidence already known, that the real digestive property of the fluid depends, not on its obvious chemical qualities, but on an organic principle. If exposed to a temperature between 104° and 122° F. or higher, it entirely and irrecoverably loses its digestive powers, although apparently, and as to analysis, unchanged. Kept from the air, the gastric fluid retains its active properties for at least two years; but, exposed to the air and a moderate temperature, it putrefies in five or six days, although the chyme which it forms from nitrogenous organic substances may be preserved for two or three months without apparent change. The precipitation of all the lime which it contains does not affect its activity; neither are its chlorides indispensable; but whatever much alters its organic constituents, (such as heat, strong alcohol, or strong acids,) or removes them, (such as animal charcoal, tannic acid, chlorine, or acetate of lead,) destroys all the digestive properties.

